# The provision of dietary and physical activity advice for men diagnosed with prostate cancer: a qualitative study of the experiences and views of health care professionals, patients and partners

**DOI:** 10.1007/s10552-017-0861-7

**Published:** 2017-02-20

**Authors:** Eileen Sutton, Lucy E. Hackshaw-McGeagh, Jonathan Aning, Amit Bahl, Anthony Koupparis, Raj Persad, Richard M. Martin, J. Athene Lane

**Affiliations:** 1 The NIHR Biomedical Research Unit in Nutrition, Diet and Lifestyle at the University Hospitals Bristol NHS Foundation Trust and the University of Bristol, Upper Maudlin Street, Bristol, BS2 8AE UK; 2 School of Social and Community Medicine, University of Bristol, Canynge Hall, 39 Whatley Road, Bristol, BS8 2PS UK; 3 Newcastle upon Tyne NHS Hospitals Foundation Trust, Freeman Hospital, Freeman Road, Newcastle upon Tyne, Tyne and Wear, NE7 7DN UK; 4 Bristol Haematology and Oncology Centre, Horfield Road, Bristol, BS2 8ED UK; 5 Southmead Hospital Bristol, Southmead Road, Westbury-on-Trym, Bristol, BS10 5NB UK

**Keywords:** Prostate cancer, Dietary advice, Physical activity advice, Health care professionals, Qualitative research

## Abstract

**Purpose:**

To explore the views and experiences of health care professionals (HCPs), men diagnosed with localised prostate cancer and their partners about the provision of advice on diet and physical activity after diagnosis and treatment for localised prostate cancer.

**Methods:**

Semi-structured in-depth interviews with ten HCPs (Consultant Urological Surgeons, Uro-Oncology Clinical Nurse Specialists and Allied Health Professionals: see Table 1) and sixteen men diagnosed with localised prostate cancer and seven of their partners. Data from interviews were thematically analysed using the Framework Approach.

**Results:**

The men and their partners provided differing accounts to the HCPs and sometimes to each other concerning the provision of advice on diet and physical activity. Some men were unable to recall receiving such advice from HCPs. Factors impacting upon advice-giving included the perceived lack of an evidence base to support dietary and physical activity advice and the credibility of advice providers. The timing of advice provision was a contentious issue as some HCPs believed that patients might not be willing to receive dietary and physical activity advice at the time of diagnosis, whilst others viewed this an opportune time to provide behaviour change information. Patients concurred with the latter opinion.

**Conclusions:**

Men and their partners would value nutritional and physical activity advice from their HCP, after a localised prostate cancer diagnosis. Men would prefer to receive this advice at an early stage in their cancer journey and may implement behaviour change if the received advice is clear and evidence-based. HCPs should receive suitable training regarding what information to provide to men and how best to deliver this information.

## Background

Prostate cancer is the second most common cancer in men worldwide, with an estimated 1.1 million cases diagnosed in 2012; almost 70% of cases occurring in more developed regions [1]. International comparisons of survival are difficult as detection of indolent cancer by prostate-specific antigen (PSA) testing causes lead time bias [2]; however, estimates show that around 84% of men in England and Wales survive for more than 10 years following diagnosis (age-standardised net survival) [3], whilst the relative 10 year survival rate in the United States (US), when including all stages of prostate cancer, is 98%. Lifestyle factors, such as poor diet, obesity, and lack of physical activity, have been identified as contributing to overall cancer risk and disease progression [4]. There is weak evidence from randomised controlled trials (RCTs) that some diet and physical activity interventions might beneficially effect prostate cancer progression [5–7], and observational studies suggest that adherence to prostate cancer-specific dietary or physical activity recommendations, such as increased consumption of plant foods or tomato products [8], or engaging in brisk walking [9], may be associated with a decreased risk of prostate cancer. However, evidence is limited. Research in the United Kingdom (UK) has illustrated that around a third of men make spontaneous changes to their diet following diagnosis and treatment, such as increasing consumption of tomatoes and fruit/vegetable juice [10] or reducing alcohol consumption [11], as well as some making changes to their physical activity levels following a prostate cancer diagnosis [11]. A prospective study in Denmark found that men diagnosed with cancer significantly decreased their tobacco and alcohol consumption and BMI between baseline and follow-up [12]; but evidence from the US suggests that changes to diet and physical activity may be short-lived [13]. Findings from the English Longitudinal Study of Ageing additionally concluded that cancer survivors were less likely to be physically active and more likely to be sedentary than those never diagnosed with cancer [14].

Some men with an increased risk of prostate cancer may be confused by conflicting health-related messages in the media and may value the provision of advice, tailored to their needs from a trusted source, such as their health care professional (HCP) [15]. The diagnosis of prostate cancer may act as a ‘teachable moment’, described by some as an opportunity to motivate individuals to spontaneously adopt risk reducing behaviours [16], so the period following diagnosis may be an opportune time to engage with patients and implement diet and physical activity interventions [17]. HCPs are well-positioned to provide advice on favourable health behaviours, but fewer than half of cancer specialists in the UK routinely discuss exercise with their patients [18] and men are rarely given dietary advice after their prostate cancer diagnosis [19]. Services to support men with prostate cancer may, therefore, need to be in place to help them implement lifestyle changes [20].

During feasibility and acceptability work for the design of a dietary and physical activity intervention for men with localised prostate cancer undergoing active treatment, we examined what diet, physical activity, and lifestyle advice were currently provided by HCPs to men, following a diagnosis of prostate cancer. In addition, we wanted to investigate how this advice was perceived by men and their partners. We, therefore, explored the opinions about, and experiences of, HCPs, men with prostate cancer, and their partners on the provision of dietary and physical activity advice following diagnosis of, and treatment for, prostate cancer.

## Methods

### Study participants

Ten HCPs (6 female, 4 male) (Table 1) directly involved in the care of men with prostate cancer, sixteen men who had been diagnosed with localised prostate cancer, and seven of their partners (Table 2) participated in in-depth semi-structured interviews. HCPs were recruited by means of convenience and snowball sampling from the team providing specific prostate cancer care at tertiary referral hospital urology department in the south-west of the UK. Table 1 outlines the HCPs specific roles. Recruitment of men was facilitated through the same hospital department with consecutively eligible men who had recently undergone radical prostatectomy, or who were undergoing radiotherapy, for localised prostate cancer, being informed about the study and invited to participate by a member of the clinical team. Those men agreeing to take part were then introduced to the study researchers.

**Table 1:** Health care professionals interviewed

Identifier prefix	Role
US (*n* = 3)	Consultant Urological Surgeon
CNS (*n* = 3)	Uro-Oncology Clinical Nurse Specialist
AHP (*n* = 4)	Cancer Support Worker/Dietitian/Physiotherapist

Consultant Urological Surgeons work specifically with men with prostate cancer. Those participating in the current research would consult with patents both prior to and following surgery as an outpatient, as well as conducting the surgery themselves

Uro-Oncology Clinical Nurse Specialists are nurses with specialist skills, knowledge, and experience in urological cancer care (including prostate cancer). Those participating in the current research provide specialist support and information to patients and their families throughout their cancer pathway

Cancer Support Workers/Dietitians/Physiotherapists are specialists, who for the purpose of this research provide advice and support to those affected by prostate cancer, for example, discussing general diet, levels of physical activity at all stages of the cancer pathway, and others, and can provide other information, such as information about community support or claiming benefits

**Table 2:** Unique identifiers and key individual characteristics of the men with prostate cancer and their partners

Identifier	Age	Relationship/civil status	Treatment	Duration since treatment at time of interview
Patient1-SUR	75	Married	Surgery	5 Months
Patient2-SUR and Partner1-SUR	70 and 63	Married	Surgery	3 Months
Patient3-SUR	67	Divorced	Surgery	12 Months
Patient4-SUR and Partner2-SUR	NK and 73	Married	Surgery	13 Months
Patient5-SUR	75		Surgery	15 Months
Patient6-RAD and Partner3-RAD	68 and 66	Married	Radiotherapy	Undergoing treatment
Patient7-SUR	53	Divorced	Surgery	3 Months
Patient8-SUR	71	Married	Surgery	3 Months
Patient9-SUR	62	Married	Surgery	2 Months
Patient10-SUR and Partner4-SUR	60 and 66	Married	Surgery	3 Months
Patient11-SUR	65	Married	Surgery	8 Months
Patient12-SUR	67	Married	Surgery	6 Months
Patient13-RAD and Partner5-RAD	79 and 77	Married	Radiotherapy	2 Weeks
Patient14-RAD and Partner6-RAD	66 and NK	Married	Radiotherapy	Waiting for treatment
Patient15-RAD and Partner7-RAD	59 and 47	Living with partner	Radiotherapy	Undergoing treatment
Patient16-SUR	67	Married	Surgery	9 Weeks

All participants diagnosed with localised prostate cancer

### Procedures and data collection

Semi-structured in-depth interviews with the men and their partners were conducted by LHM and ES between July and December 2013. Men were interviewed individually (*n* = 11) or together with their partner (*n* = 5); the majority of interviews (*n* = 16) were conducted face-to-face at the participants’ homes or in a private room at the urology clinic; some men (*n* = 7) chose a telephone interview. Interviews lasted between 26 and 97 min (mean = 49 min). All the interviews with HCPs were conducted by LHM and these lasted between 20 and 75 min (mean = 39 min).

Topic guides informed by a review of relevant literature were devised and acted as a guide for researchers during the interviews. Topics for discussion in the patient and partner interviews included experience of receiving advice and influences on making changes to behaviour following receipt of advice; the topics for discussion in the HCP interviews included the provision of dietary and lifestyle advice and factors influencing the advice dispensed. All participants were given the opportunity to articulate their views and experiences and to discuss the issues most important to them during the interview process, without being restricted by the topic guide.

Ethical approval was granted by the North West Lancaster NRES Committee (13/NW/0028) and informed consent was obtained from all individual participants included in the study.

### Analyses

Interviews were digitally recorded and transcribed verbatim. Thematic analysis of anonymised interview transcripts was carried out utilising the Framework Approach [21] (LHM, ES) with the aid of the NVivo software analysis programme. This method of analysis consists of five stages: familiarisation; identification of thematic coding framework; indexing; charting; mapping and interpretation. Each participant was given a unique identifier; in addition, men with prostate cancer, and partners, received an identifier representing the treatment type (Tables 1, 2).

## Results

Here, we present key themes arising from data analysis: health care professionals’ (HCP) views on advice-giving; the views of men and their partners on the role of HCP in providing dietary and physical activity advice; the perceived evidence base for dietary and physical activity interventions; the perceived necessity for behaviour change; the credibility of advice providers and acceptability of different resources; and the optimal timing of advice provision.

### Health care professionals views on giving generic and specific lifestyle advice

The majority of HCPs, including a dietitian, reported that they provided general rather than prostate cancer-specific dietary advice. This included drawing men’s attention to guidelines for healthy eating from the UK Department of Health (DoH) or providing them with publications from registered cancer charities which included such advice:

We are making sure they are getting the right amount of nutrients and the right balance of nutrition. You would base that on the Department of Health guidelines and like the ‘Eatwell Plate^1^’. You would just make sure all the proportions are right. (AHP3)

I’d advise them to just follow a normal healthy diet, you know to increase their amount of fruit and vegetables, cut back on fatty foods, erm, but actually I’d usually say to them, there isn’t any restriction and they don’t need to change what they are doing because you know it probably won’t impact unless somebody is overweight … (CNS2)

I give them leaflets like *Living with Prostate Cancer* and prostate cancer charities. So by and large I leave them to read the information. Give them a generic sort of attention to health and exercise … (US2)

Although the physical activity advice that was provided was reported as aligning with UK DoH guidelines, it was recognised that advice should be tailored to the individual patient’s pre-treatment level of fitness, additional health or mobility problems, treatment received, and their stage in the treatment process:

I’d encourage them to do moderate exercise, you know, walking 20 min briskly, 3 or 4 times a week, that sort of thing … especially recovering from surgery, it’s a good part of recovery anyway to get back to normal activity. (US1)

I’d normally go through what they do now, what they did before. We recommend building it up in a more step wise route, particularly for weight management. You encourage them to start with the way they are now, and work it into their day. We say, “We expect 30 min five times a week.” I think … we use standard recommendations but we tailor it more. People are so different and they start at different levels, and they’ve obviously got other things going on as well and fitting it into your lifestyle. (AHP2)

One of the Allied Health Professionals (AHPs) interviewed highlighted a perceived personal lack of expertise to provide advice on physical activity, in part because this was an area in which they were not specifically trained; whilst others talked about the importance of framing physical activity advice in a way that did not intimidate men, in particular those who did not exercise regularly:

I would get them to get it [exercise programme] verified by their consultant because I would not want to say when to start exercising. Once that has been confirmed, then I will help direct them to different areas they can go to, or different classes, different gyms or walking, but make sure they are involved with a consultant and physio. I am more going from the aspect of “Exercise is good for your overall health”, but I leave when they can do that and what type up to the experts. We are not qualified. (AHP3)

So the *[Prostate Cancer*/*Lifestyle]* 2 talks are very much about physical activity and daily routine. So things like doing the stairs, doing simple exercises at home, playing with the kids, playing with the grandkids, walking to the shops. That’s activity. So things that they may not perceive as exercise, but you’re actually saying, “Well, actually, no, that is.” (AHP1).

Some of the AHPs, including a Cancer Support Worker, in addition to a Clinical Nurse Specialist (CNS) saw part of their role as signposting patients to relevant resources, referring them to other services, or inviting them to Prostate Cancer/Lifestyle advice sessions organised at the hospital:

Probably wouldn’t recommend; if patients asked me, I would just point them, signpost them in the direction of an article so they make their own judgement and decisions. That’s the same with the research really. You can only provide as much information as you know …. (CNS3)

If they identified nutrition was a problem—it might be that we could just talk about—go through the [cancer charity] booklet together and talk about it. If it was quite an issue … we would make an appointment for that patient with the dietician. (AHP4)

A number of the HCPs interviewed explained that the levels of dietary and physical activity advice they provided were determined by perceived individual patient needs and that men themselves, or their partners, were often the catalyst for discussions on lifestyle, with some asking for prostate cancer-specific advice. The patients’ socio-demographic factors were also perceived by HCPs to have an impact upon who sought advice:

I give general advice on a prostate cancer diagnosis, because it will usually come up in the conversation. Usually patients will ask, “What can I do?” …. That’s not uncommon, actually, if they are educated and they are enthusiastic. Usually prostate cancer patients come with a file, and they will also … Internet printed out things. “I read about this. What about this treatment? What about HIFU? What about this research study? What else can I do?” For those patients we always end up discussing diet. (US3)

If they brought it up themselves it is kind of like a memory jog. I feel equipped to actually go through a lot of the data on diet and exercise. (US2)

Notably, some HCPs reported a belief that it was their role to provide targeted lifestyle advice, for example, if a patient was overweight or obese, in particular if that was having a negative impact on prostate symptoms. Then diet, physical activity, or weight loss might be discussed:

… [if] somebody is overweight and they need to lose weight before they can have surgery, or if its impacting on their urinary symptoms or something you might say actually, well losing some weight might actually be beneficial for you … (CNS2)

The only guidance I would give is as in if they have a high fat, high carbohydrate diet, I tell them to not have that, to have a balanced diet. I would also tell them to do walking, or refer them to their general practitioner to get an exercise programme. (US3)

Sometimes, the advice involved the use of “shock tactics” in an attempt to engender lifestyle change:

… it can be difficult to know, you see them once or twice and sometimes you have to say look, you know, this cancer, this could kill you, you have a better chance of not being dead in five years if you change how you are living, sometimes I say that to people … Yeah, get a grip… (US1)

### Men diagnosed with prostate cancer, and their partners’ views and experiences of the role of health care professionals

The men provided differing accounts of their experiences of receiving advice including being offered a place at a Prostate Cancer/Lifestyle event, which includes interactive lectures on diet and physical activity, or being given leaflets by their health care team:

Well they said do a little bit a walking, to keep moving a bit, keep moving, but no strenuous, no strenuous activity. (Patient1-SUR)

The only thing I did was, like they said, don’t drink tea and coffee. I didn’t drink tea and coffee, after surgery for a while. (Patient8-SUR)

Well I think they stressed it at the [Prostate Cancer/Lifestyle] day, you know, that I don’t eat enough veg. I know that, but I don’t particularly like veg. (Patient6-RAD)

Most of those who had attended a Prostate Cancer/Lifestyle session reported that they had found the advice helpful, with two participants claiming to implement dietary changes—increasing fruit and vegetable consumption—as a result of attendance. Some of the men deliberately sought out advice following diagnosis:

… they gave me all the books, and I read the books. I went on the Internet, and read what I found on the Internet, but I don’t believe a lot of what is said on the Internet. (Patient2-SUR)

… as soon as I was diagnosed I sent away with [cancer charity]… and I’ve got a great pack on what to expect you know, on your lifestyle and what to expect after it, and these, these sorts of things. (Patient9-SUR)

Some of the men could not recall being provided with any specific advice on diet and lifestyle:

I’m a little bit disillusioned by it all, because no one at any stage, the day I was diagnosed, to this day now has mentioned diets … not a sausage. Not anyone, not doctors, not nurses, not anyone. (Patient7-SUR)

Nevertheless, it was acknowledged by some that it was difficult to recall information provided around the time of diagnosis and that having a partner, or other supporter, to attend hospital appointments was helpful:

I don’t think there was much said really about diet or, or physical activity. I, I, I’m just trying to think back. I don’t want to paint a bad picture of them. But I, I can’t think that anybody said, “Oh, do this or do that” … sometimes they tell you so many things and you can’t always remember what they’ve said and what they haven’t said. So she’s [partner] always come with me, and she could say, “Well, they did say this. Well, they didn’t say that.” (Patient9-SUR)

A number of men and their partners indicated that they would have valued advice, or that they would have complied with such advice if they believed it would be beneficial:

Obviously if someone was to suggest that I do something that might help me in my—then I would listen to that advice and probably heed the advice, if anybody would care to give me—if somebody turned round and said, “Oh, your diet’s completely wrong.” Then I would go—I, I would take their advice and go along with it. Same with the ac-activity, they may say, “Oh, we should go do this or do that.” And then I’d give it a go, yeah. (Patient9-SUR)

Well, I mean, you’re… You tend to be in the hands of, of your consultants and the team, so if they, if they tell you something should be done for your benefit, then you’d probably do it. (Patient3-SUR)

### Factors impacting on the likelihood of lifestyle advice being given and adopted

### Lack of evidence base

The lack of a firm scientific evidence base, in particular, for the efficacy of dietary interventions, was viewed as barrier to the provision of advice by the HCPs:

It’s funny, but it’s [diet] quite often more talked about than the exercise. Although the data is there for the exercise it is not talked about as much. People would rather pop a pill than go to the gym more often. (US2)

Because a lot of the dietary stuff and things like that, the studies aren’t there. I always say things like, “Some people find it beneficial, some people don’t.” Rather than … because we don’t have the evidence. So suck it and see, is always my usual standard. (CNS1)

… essentially, once they have a prostate cancer diagnosis, and this may be controversial, but it’s my opinion that there’s very little that they can do. Studies have shown this as well. There’s very little they can do to change that diagnosis with diet. If anything, dietary interventions have to start earlier. (US3)

This contrasted with some HCPs willingness to provide other lifestyle advice, such as advice on giving up smoking, which might be perceived as having a more solid evidence base for potential benefits:

I feel quite comfortable to say to someone, you know, “stop smoking”, because that’s a, you know, we know that that’s actually going to benefit you, I suppose healthy eating isn’t, I don’t wanna be too prescriptive with what people can eat. (CNS2)

Some men with prostate cancer similarly emphasised the importance of scientific evidence to support any advice provided, indicating that this would aid their compliance to suggested changes, particularly in relation to their diet:

I suppose if you’ve got a, a definite link between; “If you eat that, then…” You know, in 10 years’ time, such and such might happen, then, then obviously it makes you think. (Patient3-SUR)

Nevertheless, in terms of the consumption of what they understood to be prostate cancer-specific foods, some men were unconvinced of their efficacy in disease prevention:

You read any of these health things and, [magazine], they, they say that, Saw Palmetto, and stuff. But so I was a bit shocked, I thought, “I’ve religiously taken all of these things all over the years and I used to take cod—I always take cod liver oil, well, fish oil tablets. I used to take those. I thought, “Well, I’ve taken all this and I’ve ended up with it.” (Patient9-SUR)

### No requirement for change

A further barrier to men making dietary changes was the belief that they already had a well-balanced diet, with some reporting having already made changes to their diet, for example, after experiencing heart problems or a stroke. Others believed that dietary changes would not be necessary after surgery as their prostate had been removed:

All the way through, I’ve eaten exactly the same as I would normally. A typical day for me is porridge in the morning, piece of toast, wholemeal bread. Lunch is usually a sandwich, fruit, yoghurt. In the evening it’s whatever, a cooked meal … How I would respond to that [being asked to make dietary changes], would be I’d listen, think about it, I’d come home, and then I might try it for two days, and think, “I’ve had enough of this.” Then, I would probably revert back to my normal way of eating. (Patient2-SUR)

Well I stopped it [fish oil supplement], because I, I’ve had the prostate removed anyway, but if, if it was found that to, to carry on that would, would help with cancer coming back then I would, I would go for it. But—at this moment I don’t—you know, there are all these things that are for prostate but I thought, “Well, if I’ve had my prostate removed then is there any sense in carrying on with them?” (Patient9-SUR)

I wouldn’t have bothered going [to Prostate Cancer/Lifestyle event] anyway to be quite frank with you … I consider it [prostate cancer] an inconvenience … and then, okay, I, I, hopefully it’s, you know, been dealt with now … and we can move on. (Patient12-SUR)

In contrast with some of the views of the HCPs, one man was sceptical concerning the evidence base to support beneficial effects of participation in physical activity in relation to prostate cancer. Other men and their partners revealed a lack of clarity regarding the distinction between day-to-day physical activities, such as DIY tasks, and what they viewed to be participation in exercise, such as going to the gym. The level and intensity of physical activity needed to achieve any benefit were also unclear to several participants and these were all issues that could impact upon men’s understanding of, and thus compliance with, advice received:

I think a lot of people go to gyms and such like and they have this wonderful idea they get fit, they get bored after a bit and do things, I’ve always. My physical activity has always been doing things, and erm, going with dogs walking, er, diving, doing things in that way, rather than just going and work [ing] on a treadmill. (Patient12-SUR)

The only thing he does is short mat bowling … but it’s interesting. The doctor wasn’t very impressed about that as physical activity. (Partner3-RAD)

The interviews also revealed additional important factors, such as fear of doing too much after surgery and issues of incontinence that impacted upon compliance with physical activity advice, particularly for men who had undergone surgery:

I’ve not gone running yet because I let by [leaked urine] too much, it’s just nonstop, it would be stupid. It’s—I try going out on, well my mate run last, week before last, and I went out on the push bike with him. And that was horrendous really, just non-stop—not like wetting myself, not big, but just letting by [leaking urine] every time you push—pedal. Non-stop, it was ridiculous. (Patient8-SUR)

### The credibility of advice providers and acceptability of different resources

Some men who had received leaflets from their health care team explained that they had found these difficult to understand, or that evaluation proved challenging, due to the volume of information accessible on both treatment and lifestyle, via either their health care team or the Internet:

Because I’m not medically minded, those [leaflets] went above my head. (Patient2-SUR)

… there is a lot of information around, and especially nowadays … you go down there [hospital] and you get a leaflet for this, leaflet for that, you know, and every process you go through you get a leaflet telling you about it … to me, I’m the type of person that I’d look at what the—what I’ve been asked or what is the intervention and just, you evaluate it no matter where it comes from. You look at the Internet and you take a lot of that with a pinch of salt. (Patient12-SUR)

For some, a trusted HCP, regardless of their role, was viewed as a credible source of information:

If they [HCP] had any ideas I’d be receptive to their ideas and take it from there. But probably the healthcare professional, I, I’d you know, take advice from, should I be offered it. (Patient9-SUR)

A research nurse or a consultant … just somebody who’s knowledgeable, who comes over confident and certain. (Partner3-RAD)

A matter of concern raised by a few of the men was the lack of credibility of advice, where they felt that the provider of the advice was not following it themselves. This was echoed by comments on the difficulties of being a good role model to patients by one of the CNSs interviewed:

I was very disappointed about a [HCP] who was obese telling [me] what to eat … if [they were] within a reasonable weight [themselves]. But if somebody presented information to me, what like they did, I just wouldn’t take any notice of it at all … I think you’ve got to practise what you preach. (Patient11-SUR)

… it’s difficult looking at your own [behaviour], cos you know, we don’t all have a perfectly healthy diet [laughter] it can be quite difficult to advise people, you know, tell people to cut something out completely, if you don’t do it yourself because it’s about choice isn’t it and it’s about giving people that information, and they can do with it what they want. (CNS2)

### Optimal timing of advice provision

The suitable timing of advice provision was discussed by participants. Some of the HCPs were cautious about providing advice on diet and physical activity at the time of cancer diagnosis or when discussing treatment options, as this was perceived as a psychologically and emotionally difficult time when men (or their partners) might feel overloaded and unable to absorb such information:

I have to be honest and say, given the pressure of time, the way I run a consultation, I deal with what’s on the patient’s agenda. So it is very much focused on what their concerns are. Then, for example, going for surgery we talk mainly about the surgery and its implications and complications. There is enough to discuss there. I think that overloading them with diet and exercise issues at that stage is not something to do and I don’t do that. Prior to surgery, no. (US2)

Nevertheless, the importance of tailoring any advice given to suit the individual was emphasised by some HCPs, with one of the Clinical Nurse Specialists explaining that men respond to advice-giving in different ways and that making changes in diet or physical activity might enable them to wrest back some control over part of their life when everything else was being dictated by HCPs:

People often feel that they have lost control when they have got a new diagnosis and they are being seen in hospital and things, so being told to change their diet or to change something could be another thing that they feel we are controlling, or maybe they didn’t have the control over, so it’s quite complex and I think people respond to things very differently. (CNS2)

In contrast to some of the HCPs’ opinions, however, most of the men interviewed indicated that they would have appreciated receiving advice at an early stage in their treatment:

I think definitely there should be definitely a programme where you’re encouraged to eat healthy. From the minute you’re diagnosed with something … it’s never too late or soon to start a healthy diet, is it? (Patient7-SUR)

There’s never a right time but it’s never too soon really. I, I, think really, erm, if, if you’re going to intervene and say that th-these are changes you should do, it should be straightaway. In fact even before surgery. (Patient12-SUR)

For some HCPs, diagnosis and pre-treatment consultations were viewed as an opportunity to speak with men about making lifestyle changes; for others, the period following treatment was the key opportunity for implementing behaviour advice:

[Diagnosis is] the teachable moment, if you like. But it is that point in your life where you actually take stock, and think, “Hang on.” (AHP1)

Well, I think exercise is really important. I think they have to be motivated, and they have to have a long-lasting effort at it. So if they only do exercise for six weeks prior to surgery, and then they never do it again, then it’s pretty pointless. I think that exercise helps on so many levels. That’s the way that I explain it to a patient. I say, “Well, it’s not just for the surgery. Your heart will be stronger. Your lungs will be better. You will generally get a better outcome cardiovascularly.” Often, as well, sometimes prostate cancer, you may hear other people involved with prostate cancer patients say this. Prostate cancer is the trigger that then triggers them to a fitter lifestyle. (US3)

… in that period of six weeks’ recovery afterwards—and it is shorter for robotic surgery, but they still have to take it easy. There is a period during which their minds might be free to actually absorb this information. It might be a good time to actually start giving them this information. Their priority is—for many people they have busy lives, it’s the only time they focus on themselves—and this could be another aspect of that … Anyone who has cancer, speaking personally, they refocus. Diet and exercise is one of those things that quite often blokes are pretty bad and they let themselves go. (US2)

As noted above, immediate concerns, such as coping with incontinence post-surgery, may, however, also need to be considered.

For the men themselves, it might be a time when they took stock of their life or were willing to make significant changes to behaviour or readdress their priorities to improve their quality of life:

I mean it’s like saying about our car, five or six years ago if somebody had come to me and said, “I’m sorry I’ve run into your car I’d get up and kill ‘em. I just sat there and they all looked at me as if, and said, “Are you alright?” [Laughter] Didn’t, didn’t bother me. … I said, “I’ve got more important things on my mind than the car.” … life, life is precious. I don’t worry about little things like cars now. (Patient10-SUR)

If somebody said to me, “You’ve got to walk from here to the ring road” which is probably five minutes, ten minutes’ walk, a fast walk, “And back every day, otherwise you’ll be dead in six months” [I] would do it without hesitation. (Patient6-RAD)

## Discussion

This research explored the views and experiences of HCPs, men diagnosed with prostate cancer, and their partners concerning the provision of diet and physical activity advice following diagnosis of, and treatment for, localised prostate cancer. HCPs indicated that they did not routinely provide advice on diet and physical activity to men diagnosed with prostate cancer, but if they did the information provided by them which was usually generic and in keeping with UK DoH guidelines for the general population. Some HCPs offered men prostate cancer-specific information leaflets published by registered cancer charities or signposted them to services, such as Prostate Cancer/Lifestyle advice days, which include presentations on diet and physical activity, or to exercise programmes run by cancer charities. Many of the HCPs demonstrated awareness that interventions need to be tailored to the individual; however, this rarely equated to tailored advice being delivered. This was particularly the case for dietary advice, where HCPs appeared to lack confidence regarding their knowledge about a prostate healthy diet. HCPs often highlighted the lack of conclusive scientific dietary evidence available, and it was their belief that any such advice should be delivered by an ‘expert’, which most did not class themselves as. The CNSs and AHPs implied that they would be happy for Consultants to dispense advice, whom they saw to be the ‘experts’. In some instances, HCPs appeared to be more confident at delivering tailored physical activity advice, although some AHPs were wary of giving advice on activity programmes, unless they were approved by a Consultant. Although this was not standard practice for all, amongst those who did provide advice on physical activity, there was a general understanding that pre-treatment level of fitness, existence of additional health or mobility problems, type of treatment received, and the duration since treatment, should be taken into consideration. Further to this, the provision of dietary and physical activity advice was not always seen to be a priority by the HCPs. Discussion on lifestyle issues was often instigated by the men or their partners—particularly those from higher socioeconomic groups—rather than by the HCP, with HCPs often commenting that if the patients, or partners, did not ask for advice, none was offered.

The men and their partners differed to the HCPs and in some cases to each other in their accounts of advice provision, with some unable to recall receiving any advice on diet or physical activity, aligning with the previous studies which have found that men were rarely given dietary advice [19] or advice on physical activity [18] by their HCP after prostate cancer diagnosis. However, other interviewees were able to provide examples of advice received, with a few reporting having made subsequent changes to their diet. The men also indicated, in line with existing research [19, 22], that they would value advice on diet and physical activity following diagnosis. The importance of receiving advice from a trusted source was highlighted as being key for the men and their partners, particularly in terms of the likelihood of them acting on such advice, aligning with the previous research which found that a majority of social network members and cancer survivors believe that it is a doctor’s duty to provide lifestyle advice [23]. This conflicted with HCPs perspectives, as they did not see themselves as being an ‘expert’ in a position to deliver this advice; however, patients welcomed it and believed that any advice provided by a HCP, especially a Consultant, was valid and worth heeding. A number of the men, and their partners, demonstrated an awareness of purported ‘prostate-friendly’ foods; however, there was some lack of belief in the efficacy of such foods in disease prevention or in reducing disease recurrence or progression. A lack of clarity concerning the definition of what counts as physical activity and the level of intensity required to achieve possible benefits was also articulated by men and their partners.

Many HCPs illustrated an opinion that the best time to provide advice was somewhat dependent on the individual patient, and as previously stated, advice was only provided when it was specifically requested. It was suggested by some HCPs that diagnosis was not always a suitable time to provide advice, as the man would already have been given vast amounts of information, and may be experiencing great distress—this was seen to be a barrier for HCPs to provide information. This equates with research findings that distress, such as that experienced when receiving a diagnosis of cancer, can impact negatively upon the uptake of knowledge [24]. Interestingly, this was the reverse of the opinions provided by patients and partners, who illustrated a distinct wish for information about diet, physical activity, and lifestyle changes from the moment of diagnosis. Thus, a barrier for some HCPs appears to be an incorrectly perceived perspective about the patients’ willingness to accept advice at time of diagnosis. This was not the case for all HCPs, however, as some noted that diagnosis was an ideal time to provide behaviour change information, as the point of diagnosis was seen as a potential teachable moment and a suitable opportunity to initiate change, which was in accordance with opinions of the patients. The concept that a prostate cancer diagnosis may constitute a teachable moment has been observed by other studies [11, 19, 25], where men might consider acting upon dietary and physical activity advice to instigate lifestyle changes. Finally, it was agreed, by HCP, patients, and partners alike, that HCPs are often seen to be ‘role models’, and thus, their own behaviour can have an impact upon the patients belief, understanding, and uptake of any information provided. An element of discomfort was noted by some of the HCPs in telling patients what to do, particularly if they were instigating behaviour that they themselves did not adhere to, such as maintaining a healthy body weight. This was reflected in the opinions of the patients and partners, who demonstrated a belief of ‘practice what you preach’ and thus would be *more* likely to adhere to advice that they believed was being followed by the HCP.

A potential limitation of this study is that all the interviews were conducted with staff, patients, and partners recruited from one hospital urology department and that access to information and advice via Prostate Cancer/Lifestyle days (or similar events) may not be available to men nationally. The HCPs interviewed had different roles and training/experience in terms of the provision of dietary and physical advice; for example, dietitians and physiotherapists might be expected to have more experience to providing such advice. However, the HCPs included in the sample were those who usually have contact with men along the prostate cancer care pathway, and this was what we had intended to capture. All the men interviewed were of White British ethnicity, thus restricting generalisability. The men interviewed had all undergone, or were in the process of undergoing, active treatment (surgery and radiotherapy) for localised cancer as the study was designed as preparatory work for the design of a dietary and physical activity intervention for men with localised prostate cancer undergoing active treatment. Men on active surveillance or those with metastatic prostate cancer were, therefore, not approached to participate. The men included in the study varied in age, level of education, and occupation status.

## Conclusions

This research has produced interesting and potentially novel findings in relation to the provision of dietary and physical activity advice that have implications for clinical and public health. A prostate cancer diagnosis may represent a teachable moment and this study has identified that the patients and their partners interviewed were ready to receive, and value, advice on dietary, and lifestyle improvement, at the time of diagnosis. HCPs, however, are often unsure of what advice to provide, especially with regards to tailored interventions, frequently have concerns regarding whether the patient would be open to receiving this advice, and may doubt their specific knowledge and ability to provide such information. They may also lack sufficient time to provide such advice within routine NHS consultations, meaning that they prioritise other aspects of clinical care. A lack of requests for advice from patients and their partners, however, should not be viewed as a lack of need for such advice. Our findings indicate that men would value such information, from the early stages of their cancer journey. The provision of advice at an early stage may help empower men to feel that they are doing something positive to aid their recovery or prevent reoccurrence following treatment. It may also assist/help men to make judgements regarding the validity of available information sources, particularly regarding dietary change. Therefore, evidence-based advice and behaviour change interventions need to be made available to prostate cancer patients, from the moment of diagnosis, to allow them to make informed decisions about their health behaviours. It is important that HCPs receive sufficient, suitable training regarding what information to provide patients with and how to go about doing so. This would empower HCPs, illustrate to them the vital importance of such advice, that it is desired by patients, which should be a priority in their consultations, and give them the confidence to provide these much needed interventions. Further randomised controlled trials are needed to better understand and identify key dietary and physical activity interventions for men with prostate cancer, so that HCPs will feel confident that the information that they are providing is gold standard and evidence-based.

## Footnotes

1 The Eatwell Plate is a UK government initiative, which highlights the different types of food that make up our diet, and shows the proportions we should eat them in to have a well-balanced and healthy diet. The USA equivalent is called ChooseMyPlate.

2 Prostate Cancer/Lifestyle events (funded and run by the hospital Trust) are half/full day of talks by a range of health care professionals, providing information and education about health and wellbeing, recovery and rehabilitation, education in self-management, signs and symptoms of recurrence, reducing risk factors, managing side effects of disease and treatment, diet and exercise, anxiety and stress management and financial advice. The aim of the event is to ensure that those living with and beyond cancer get the care and support they need to lead as healthy and active a life as possible, for as long as possible.
